# A systematic review of selected human rights programs to improve HIV-related outcomes from 2003 to 2015: what do we know?

**DOI:** 10.1186/s12879-019-3692-1

**Published:** 2019-03-05

**Authors:** Anne L. Stangl, Devaki Singh, Michael Windle, Kirsty Sievwright, Katherine Footer, Alexandrina Iovita, Stella Mukasa, Stefan Baral

**Affiliations:** 1 Department of Global Health, Youth and Development, International Center for Research on Women, 1120 20th St. NW Suite 500N, Washington, DC 20036 USA; 2 Center for Public Health and Human Rights, Department of Epidemiology, Johns Hopkins School of Public Health, Baltimore, MD USA; 3 Human Rights Division, Joint United Nations Programme on HIV/AIDS, Geneva, Switzerland

**Keywords:** Systematic review, HIV, Legal, Interventions, Human rights, Structural

## Abstract

**Background:**

Repressive legal environments and widespread human rights violations act as structural impediments to efforts to engage key populations at risk of HIV infection in HIV prevention, care, and treatment efforts. The identification and scale-up of human rights programs and rights-based interventions that enable coverage of and retention in evidence-based HIV prevention and treatment approaches is crucial for halting the epidemic.

**Methods:**

We conducted a systematic review of studies that assessed the effectiveness of human rights interventions on improving HIV-related outcomes between 1/1/2003–28/3/2015 per PRISMA guidelines. Studies of any design that sought to evaluate an intervention falling into one of the following UNAIDS’ key human rights program areas were included: HIV-related legal services; monitoring and reforming laws, policies, and regulations; legal literacy programs; sensitization of lawmakers and law enforcement agents; and training for health care providers on human rights and medical ethics related to HIV.

**Results:**

Of 31,861 peer-reviewed articles and reports identified, 23 were included in our review representing 15 different populations across 11 countries. Most studies (83%) reported a positive influence of human rights interventions on HIV-related outcomes. The majority incorporated two or more principles of the human rights-based approach, typically non-discrimination and accountability, and sought to influence two or more elements of the right to health, namely availability and acceptability. Outcome measures varied considerably, making comparisons between studies difficult.

**Conclusion:**

Our review revealed encouraging evidence of human rights interventions enabling a comprehensive HIV response, yet critical gaps remain. The development of a research framework with standardized indicators is needed to advance the field. Promising interventions should be implemented on a larger scale and rigorously evaluated. Funding for methodologically sound evaluations of human rights interventions should match the demand for human rights-based and structural approaches to protect those most vulnerable from HIV infection.

**Electronic supplementary material:**

The online version of this article (10.1186/s12879-019-3692-1) contains supplementary material, which is available to authorized users.

## Background

Our ability to ‘end AIDS’ in a generation, by ensuring that the majority of people living with HIV know their status, are on treatment, and are virally supressed and by reducing new infections, will depend largely on our success at reducing the stigma, discrimination and other human rights violations faced by key and vulnerable populations most at risk of HIV infection [1, 2]. Key populations are defined by UNAIDS to include: sex workers, men who have sex with men, transgender people, and people who inject drugs [3], while vulnerable populations, as defined by the World Health Organization, may include young people, women, migrants, prisoners or other populations whose “living conditions are prone to shifting factors which would place them at risk of contracting HIV” [4]. Stigma and discrimination related to HIV and key population status are commonly acknowledged barriers to reaching and engaging marginalized populations in HIV services [5, 6].

New data suggest that increasing coverage of HIV prevention and treatment interventions is necessary, but not sufficient in ending HIV transmission by 2030 [7]. While interventions to increase the availability and acceptability of HIV services have expanded over the last decade [8–11], interventions to increase accessibility among vulnerable and key populations are generally limited [12–14]. According to UNAIDS, expanding accessibility will depend on our collective capacity to provide people-centred, high quality health services based on universal human rights standards coupled with the repeal or reform of laws and policies to end punitive practices [15]. To this end, UNAIDS prioritized seven human rights programs for inclusion in AIDS responses, including: (1) HIV-related stigma and discrimination reduction programs; (2) HIV-related legal services; (3) monitoring & reforming laws, policies, and regulations; (4) rights and legal literacy programs; (5) sensitization of lawmakers & law enforcement agents; (6) training for health care providers on human rights and medical ethics related to HIV; and (7) reducing discrimination (e.g. gender inequality and violence) against women in the context of HIV [16]. The types of interventions that fall under these seven categories are typically structural in nature, but often include activities that address other socio-ecological levels (i.e. individual, interpersonal, organizational and community) [17, 18].

Structural interventions work to alter the social, economic and political contexts that influence individual, community and societal health outcomes [19]. There is a solid body of evidence documenting the effectiveness of structural interventions on improving public health outcomes, ranging from seat belt laws, to regulations on violence in the media, to water fluoridation [12]. Yet while 30 years of the HIV response have demonstrated the importance of empowered communities claiming their rights [20, 21], we still do not have solid evidence on the impact of structural interventions designed to reduce new HIV infections, increase HIV testing and improve health outcomes for people living with HIV.

Over the last decade, international consensus has been established on the importance of respecting, protecting and promoting human rights and incorporating the principles of a rights-based approach in the response to HIV [22]. Rooted in the established definition of human rights as universal and inalienable [23], a human rights-based approach (HRBA) seeks to keep those who are marginalized, excluded or discriminated against as the central focus when envisioning policy components and outcomes.

Programs and policies founded on a rights-based approach encourage rights holders (e.g. individuals) to claim their rights, while enhancing the capacity of duty-bearers (e.g. health care providers) to meet their obligations [24]. Regarding health, and specifically HIV, a rights-based approach involves integrating the principles of non-discrimination, participation, empowerment, accountability and linkages to other rights into the design, implementation, monitoring, and evaluation of health-related programs and interventions [25, 26]. In addition, human rights programs should also encompass the attributes of the right to health, including: availability, acceptability, accessibility and quality of health facilities and related goods and services [27].

### Previous reviews

Of the seven categories of human rights programs defined by UNAIDS, two have been studied extensively. Interventions to reduce HIV-related stigma and discrimination have been reviewed previously by Brown et al. [28], Sengupta et al. [29] and Stangl et al. [18]. Collectively, these reviews identified key intervention categories and documented strong evidence on effective approaches to reduce HIV-related stigma and discrimination. Reducing discrimination affecting women in the context of HIV has also been studied previously, including systematic reviews documenting interventions to improve gender relations, increase women’s control over household assets and reduce gender-based violence [30–32]. Given existing evidence on these two human rights program categories, we focused the current review on the five remaining categories (see Additional file 1: S1).

### Current review

While human rights programs have great potential to improve equity and access to HIV prevention, care and treatment services [16], little is known about their effectiveness at improving HIV-related outcomes. In the present review, we examined two categories of HIV-related outcomes: (1) human-rights related barriers to HIV services, and (2) HIV prevention care and treatment outcomes. Human rights-related barriers that inhibit access to, uptake of and adherence to HIV services among key and vulnerable populations have been well documented [33, 34]. Examples include stigmatizing sex workers and men who have sex with men in health facilities [35, 36], illegal policing practices that target people who inject drugs [37], and lack of knowledge and implementation of harm reduction policies [38]. These barriers indirectly influence HIV-related outcomes by discouraging engagement in prevention, care and treatment services [39, 40]. HIV prevention, care and treatment outcomes assessed ranged from HIV risk behaviors, to uptake of HIV testing, to experience of gender-based or intimate partner violence, to HIV incidence.

The paucity of rigorous evidence makes it difficult for governments to integrate human rights programs within a comprehensive response to HIV [41]. To fill this gap, we systematically reviewed peer-reviewed and grey literature. Our goal was to ascertain the human rights interventions implemented, their influence on HIV-related health outcomes, the socio-ecological levels addressed, the attention paid to the attributes of the right to health, the incorporation of the principles of a human rights-based approach, and the study quality.

## Methods

### Search strategy and selection criteria

This review followed PRISMA guidelines. Our search strategy involved three expansive search components for each database, including: (1) legal terms, defined broadly to include any references to rights or structural interventions, among other terms; (2) population terms, including HIV and related vulnerable and key populations; (3) and evaluative terms. These were adapted as appropriate for each database (see Additional file 2: S2). Articles were pulled if they matched on at least one term in each of the three search components. PubMed, Scopus, Embase, Ovid Global Health, Sociological Abstracts, PAIS International and Popline were searched for peer reviewed literature. Scopus, Popline, and PAIS International were also searched for grey literature. Additional grey literature was obtained from the https://www.aidsactioneurope.org//en/clearinghouse, USAID Development Experience Clearinghouse, UNESCO HIV and AIDS Education Clearinghouse, Google, WHO and UNAIDS. References were exported to EndNote X7 (Thomson Reuters) and de-duplicated. The title, author, journal and year of publication were then exported to an Excel spreadsheet for title review. Ancestry searches reviewing the citations of the 23 articles included in the review were also conducted.

Inclusion criteria included an evaluation design (pre- and post-test data or post-test data only), or an analysis framework articulated for policy reviews, clear descriptions of the intervention and publication in English. We limited our search to articles published between January 1, 2003 and the finalization of the search protocol on March 28, 2015. 2003 was selected as the starting point for the search as this was the year the United Nations Development Group adopted the UN Statement of Common Understanding on Human Rights-Based Approaches to Development Cooperation and Programming [26], referred to as the ‘the Common Understanding’. Studies of any design from any country that included HIV-related health outcomes and addressed one or more of the five UNAIDS’ human rights programs of interest were included [16].

We did not exclude studies that lacked a clear description of the sampling strategy or measures used, or studies that did not explicitly describe the intervention under study as a ‘human rights intervention’, due to the nascent stage of research in this field. The decisions for inclusion and exclusion of manuscripts were made based on a priori criteria and completed independently by two reviewers with tiebreakers by a third. As part of this process, three studies that assessed the influence of policies mandating the offer of opt-out, provider-initiated HIV testing were included [42–44]. While opt-out testing has been contentious since its introduction in 2007 [45], these policies are often recommended by national and global health agencies, like the U.S. Centers for Disease Control and Prevention and the World Health Organization, as well as national and state governments, and thus require scrutiny from a human rights perspective.

### Screening and data abstraction

Article citations were organized, uploaded and reviewed from their respective databases. Each title was reviewed by two of four reviewers (KS, DS, MW, AS) to determine whether they included relevant information [46]. If an article was deemed relevant by one reviewer, the abstract was retrieved for review. All abstracts were then reviewed by two reviewers (KS, DS) to determine their relevance. Discrepancies were discussed with a third senior reviewer (AS), and consensus was reached on whether to include the article. Each full text was reviewed by two of six reviewers (KS, DS, AS, KF, MW, AI). For the selected studies, data were abstracted using a standardized abstraction form (see Additional file 3: S3). Validity of HIV-related outcomes measured in the quantitative studies was not examined, as none of the studies utilized scales assessing latent constructs (Table 1).

**Table 1:** Study and intervention characteristics, HRBA elements incorporated, and study findings from 23 studies

1st Author, publication date, country, study design	Study Population	Sample	Human Rights Program Category; Intervention Duration	Socio-ecological Levels; Right to Health Attributes	HRBA Principles; Implicit /Explicit^A^	HIV-related Outcomes	Results (Positive, Negative, No effect; Details)
	*Evaluation Studies*						
Argento, 2011 [63], India, Qualitative post-test only	Sex workers, LGBTQ^B^, Police, General population	34 SWs; 12 key informants^C^	Sensitization of law makers and law enforcement agents; Not specified	Community; Availability, Accessibility, Acceptability	Accountability, Linkages/Implicit	Violence against women (VAW),, human rights violations	Positive; Strategies to combat violence against women among sex workers developed; violence from the police reduced; remaining challenges: risk of violence from regular partners who do not want to use condoms.
Beletsky, 2011 [59], USA, Observational repeated cross-sections	Police	94	Legal literacy, Sensitization of law makers and law enforcement agents; One, 30-min session	Individual; Accessibility, Acceptability, Quality	Empowerment/ Implicit	Knowledge of harm reduction programs, health risks from needle stick injuries	Positive; Trainee’s knowledge increased; no significant increases in attitudes.
Beletsky, 2013 [65], Kyrgyzstan, Observational cross-section	Police	313	Legal literacy, Sensitization of law makers and law enforcement agents; 46-h “Special Course” on harm reduction for senior officers; ad-hoc shorter modules delivered to new police trainees	Individual; Accessibility, Acceptability, Quality	Empowerment, Non-discrimination, Participation, Accountability, Linkages/ Implicity	Legal and policy knowledge of PWID and SW, opinions about referral to harm reduction, intent to confiscate syringes	Positive; Trainees more likely to: support referral to harm reduction; indicate no intent to confiscate syringes; understand sex worker detention policies and procedures after occupational exposure. Knowledge of policy on routine ID checks for sex workers significantly lower for trainees.
Beletsky, 2012 [66], Kyrgyzstan, Observational cross-section	Police	319	Legal literacy, Sensitization of law makers and law enforcement agents; Instruction 417 promulgated in 2009	Policy; Accessibility, Acceptability, Quality	Non-discrimination, Participation, Accountability/ Implicit	Knowledge and attitudes towards: harm reduction, due process for detention of SWs, occupational exposure, past and intended future practices with vulnerable groups	Positive; Knowledge of Instruction 417^D^ assoc. with better knowledge and attitudes towards harm reduction and due process for detention of sex workers. Younger, junior officers and those in rural areas may not be well informed about the policy.
Du 2011 [42], USA, Observational cross-section	General population (aged 18–64)	281,826	Monitoring and reforming laws, policies and regulations; CDC “opt-out” HIV testing strategy recommended in 2006	Policy; Availablity, Accesibility	Not applicable	Ever tested for HIV	Positive; Compared with residents of “high morbidity-opt out” states, those living in “high morbidity-opt in” and “low morbidity” states had lower odds of being tested for HIV.
Dworkin, 2014 [55], Kenya, Qualitative post-test only	Women	50	HIV-related legal services, legal literacy; GROOTs-Kenya developed the “Community Land and Property Watch Dog model” in 2005	Community; Availability, Accessibility, Acceptability	Non-discrimination, Accountability/ Implicity	Women’s access to and ownership of land	Positive; Local mediation has greater success than formal court system in securing women access to their land; community-level solutions important for responding to property rights violations.
Ellen, 2015 [60], USA, Observational repeated cross-sections	At-risk Youth	2559 (aged 12–24)	Monitoring and reforming laws, policies and regulations, Sensitization of law makers and law enforcement agents; the Connect to Protect project ran 2002–2012, data collected 2007–2010	Organizational, policy; Availabiltiy, Accessibility	Empowerment, Accountability/ Implicit	No. of sexual partners, partner characteristics, condom use, and history of sexually transmitted infections and HIV testing	No effect; Exposure to structural changes was not statistically significantly associated with any of the outcome measures.
Fang, 2004 [64], Taiwan, Observational repeated cross-sections	General population	Not applicable^E^	Monitoring and reforming laws, policies and regulations; Free HAART access implemented in 1997	Policy; Availability, Accessibility, Quality	Non-discrimination/ Implicit	HIV transmission rate, incidence of syphilis and gonorrhea	Positive; After free access to HAART was established, the estimated HIV transmission rate decreased by 53%. There was no statistically significant change in the incidence of syphilis.
Grangeiro, 2011 [67], Brazil, Observational repeated cross-sections^F^	General Population	812 municipalities	Monitoring and reforming laws, policies and regulations; MOH strategies for municipalisation of the response to AIDS first implemented 1994–2002 with the second phase launching in 2003	Policy; Availability, Accessibility, Acceptability, Quality	Empowerment, Non-discrimination/ Implicit	HIV testing, HIV incidence by key populations, presence of AIDS services, prevention coverage, mortality	Positive; Municipalities included from 1994 to 1998 showed higher chances of providing HIV diagnostic testing, of having AIDS services, and reducing cases involving heterosexual, homosexual/bisexual, and IDU transmission as compared to those “included in 2003” and “not included”. Municipalities with a more structured response were associated with better results.
Gruskin, 2013 [25], Kenya, Qualitative post-test only/secondary analysis of program data	PLHIV^G^; Survivors of gender-based violence	IDIs (no. NS) and FGDs (no. NS), with legal and health staff, patients, and clients. Review of legal aid records (450/LACE), and client cases (73/COVAW)and 18/CHAK).	HIV-related legal services, Legal literacy, Training of health care providers on human rights and medical ethics; three legal intervention programmes, earliest indicated start of one of these programmes was 2007	Community; Availability, Accessibility, Acceptability, Quality	Participation, Accountability/ Explicit	Access to and utilizations of health services among PLHIV and survivors of violence	Positive; Increases in client’s practical knowledge and awareness about how to access legal aid and claim their rights, to communicate with healthcare providers and to improve their access to healthcare and justice. In turn, providers became more adept at identifying human rights violations and other legal difficulties, which enabled them to better support clients.
Homaifar, 2005 [56], Senegal, Observational cross-section	Sex workers	60	Monitoring and reforming laws, policies and regulations; Senegal legalized the practice and registration of all sex trade workers in 1969	Individual, policy; Availability, Accessibility	Non-discrimination/ Implicit	Knowledge of sexual health, circumstances of daily life, sexual history, patient knowledge of contraceptives and STIs, condom use, risk of contracting HIV	Positive; Increased knowledge of health, adamant in contraceptive use, desire to put health before money, demand use of condoms, due to the registration requirements and increased knowledge lower chances of putting themselves in HIV risky situations
Jardine, 2012 [49], Vietnam, Qualitative post-test only/Observational cross-section/secondary analysis of program data	Police	36 mixed KIIs 27 police surveyed	Monitoring and reforming laws, policies and regulations, Sensitization of law makers and law enforcement agents; in 2006 Vietnam instituted the HIV/AIDS Law which included comprehensive harm reduction measures	Organizational; Availability	Non-discrimination, Accountability/ Implicit	Police knowledge and attitudes about harm reduction; police actions towards PWID in the context of harm reduction	No effect; Police still target PWID and engage in contrary activities; lack knowledge of harm reduction (in the context of police training, laws and policies); don’t support NSP; lack national training for ward police in harm reduction
Jones, 2005 [57], South Africa, Qualitative post-test only	PLHIV, non-PLHIV (i.e. members of social clubs, youth groups, local govt. councillors, health care workers, NGO volunteers)	FGDs (no. NS), KIIs (no NS), clinic observations (no. NS)	Monitoring and reforming laws, policies and regulations; NS	Policy; Availability, Acceptability	Non-discrimination/ Explicit	Knowledge and understanding of human rights as they relate to PLHIV, ability to access rights if needed	No effect; Participants are still subject to HR violations, such a breach of confidentiality, forced HIV testing, denial of or poor quality health care services, etc. Fear of stigma keeps many people from disclosing their status. Little to no impact of the national policies and laws in place to protect the rights of PLHIV.
Martinez, 2007 [61], USA, Observational repeated cross-sections	People who inject drugs	1578	Monitoring and reforming laws, policies and regulations; Legislation passed in 2000 allowed SEPs in CA	Policy; Availability, Accessibility	Non-discrimination, Empowerment, Participation/ Implicit	HIV testing, impact on PWIDs (overall arrest, arrest or citation for drug paraphernalia, etc)	Negative; Legal SEPs increased odds of clients being arrested or cited for drug paraphernalia
Rich, 2007 [62], USA, Observational repeated cross-sections^F^	People who inject drugs	473	Monitoring and reforming laws, policies and regulations; Rhode Island passed legislation in 2000 completely legalizing the sale of non-prescription syringes by pharmacists	Policy; Availability	Accountability, Non-discrimination/ Implicit	Syringe use of PWID (frequency, whether or not it was used, etc)	Positive; Those with legal access were more likely to utilize pharmacies as a source of sterile injection equipment.
Sarnquist, 2007 [43], USA, Observational repeated cross-sections	Pregnant Women	496	Monitoring and reforming laws, policies and regulations; 1995 California law mandating an HIV test and treatment offer to every pregnant women	Policy; Availability, Accessibility	Not applicable	Offers of HIV testing and treatment	Positive; No. of offers of HIV testing and treatment significantly improved
van Rensburg, 2007 [58], South Africa, Observational cross-section	PLHIV, Survivors of violence	304	Legal literacy; Lifeline Opened in 1971	Community; Availability, Accessibility	Non-discrimination, Participation/ Implicit	GBV and HIV knowledge awareness, attitudes, GBV and HIV risk factors, behaviour changes, disclosure, availability and accessibility of support and care, relationships and condom use	Positive; GBV and HIV awareness/knowledge levels were high, perceived GBV is high as is perceived risk; positive changes in behaviour were noted to prevent HIV; high incidence of women being subjected to GBV; difference noted between age, older respondents less likely to know legal rights.
	*Policy Reviews*						
Ahmad, 2013 [51], South Africa, Policy review	PLHIV, Pharmaceutical companies, General Population	N/A	HIV-related legal services, Monitoring and reforming laws, policies and regulations, Legal literacy; TAC was established1998	Individual, organizational, community; Availability, Accessibility	Accountability, Empowerment, Non-discrimination, Participation/ Explicit	Access to ARVs	Positive; Efforts of the Treatment Action Campaign resulted in access to nevirapine in public hospitals, reinforcement of the decision that led to access to ARVs in prison, Pfizer committing to supply free-of-charge fluconazol to public healthcare clinics and PMA withdrawing claim against Government, allowing the Government to reduce essential drugs prices.
Ainsworth, 2003 [52], Thailand, Policy review	Sex workers (primary), Clients of sex workers, General population (focus on young men)	NS	Monitoring and reforming laws, policies and regulations,,, Sensitization of law makers and law enforcement agents; Thailand’s policy response to AIDS prior to 2000	Policy; Availability, Accessibility	Empowerment, Non-discrimination, Accountability, Participation/ Implicit	Condom use, risk sexual behaviour, reported STD cases, HIV prevalence	Positive; Change from punitive legal and policy environment to rights-based. Condom use in brothels rose significantly; the No. of reported STD cases declined while the No. of STD clinics grew and drug stores reported decrease in sale of antibiotics for STD and increase in sale of condoms; HIV prevalence among 21-year old army conscripts decreased.
Gruskin, 2009 [53], 133 Countries, Policy review	Vulnerable groups^H^	133 United Nations General Assembly Special Session Country Progress Reports	HIV-related legal services, Monitoring and reforming laws, policies and regulations; in 2001 the United Nations General Assembly Special Session (UNGASS) Declaration of Commitment on HIV/AIDS (DoC) emphasized the centrality of human rights to an effective HIV response.	Policy; Availability, Accessibility	Participation, Accountability/ Explicit	Legal environment barriers to access to HIV services	Positive; Increase in the No. of countries reporting on human rights issues between 2006 and 2008. 94% of reporting countries note that their national HIV policies explicitly mention the promotion and protection of human rights, yet only 22% of these countries report performance indicators to assess human rights compliance.
Gurnani, 2011 [54], India, Program monitoring data	Sex workers, Police, Lawyers, Media	Used data from 18 Districts where over 900 peer educators are employed and an average of 50,000 FSWs are contacted per month	HIV-related legal services, Monitoring and reforming laws, policies and regulations, Legal literacy, Sensitization of law makers and law enforcement agents; Karnataka Health Promotion Trust was established in 2003 with the aim of rapidly scaling up HIV prevention efforts	Individual, community, organizational; Availability, Accessibility, Acceptability, Quality	Non-discrimination, Participation/ Explicit	Reduction in HIV risk, stigma and discrimination, violence	Positive; FSW membership in community-based organisations has notably increased and over 46,000 FSWs have now been referred for government-sponsored social entitlements. FSWs were supported to redress > 90% of the 4600 reported incidents of violence and harassment reported between 2007 and 2009, and monitoring of news stories has shown a 50% increase in the No. of positive media reports on HIV and FSWs.
Jeffreys, 2011 [50], China, Policy review	Sex workers	NA	Monitoring and reforming laws, policies and regulations, Sensitization of law makers and law enforcement agents; China’s Ministry of Health (MOH) and the WHO launched a pilot 100 Per Cent Condom Use Program in 2001	Policy; Availability, Accessibility	Empowerment, Accountability/ Implicit	Rates of Chlamydia infection, HIV prevalence, local condom sales in the pilot sites	Positive; PRC MoH estimates that health interventions had only managed to reach 40% of SWs and MSM by the end of 2009. It further estimates that only 41% of FSWs use condoms consistently (increase from baseline of 14.7% in 2001) and that fewer than 30% of men who have sex with men use condoms
Lazariu, 2015 [44], USA, Program monitoring data	General population (receiving health services)	166	Monitoring and reforming laws, policies and regulations; in 2010, New York State (NYS) Public Health Law mandates the offer of HIV testing to all persons aged 13–64 years receiving hospital or primary care services	Policy; Availability	Not applicable	HIV testing	Positive; HIV testing volume increased by 13% following enactment.

^a^Explicit/Implicit: denotes whether a human rights-based approach was explicitly stated by the researcher or implicit in described approach; ^B^ LGBTQ = lesbian, gay, bisexual, transgender, and questioning; ^C^12 interviews conducted with transgender sex workers, police officers, brokers, boyfriends and lodge owners; number of interviews per group not specified. 2 focus group discussions were also conducted, one each with male and female sex workers; number of participants not specified; ^D^ Instruction 417 was disseminated by the Kyrgyz government prohibiting police interference with “harm reduction” programs, re-enforcing citizen rights, addressing police occupational safety concerns, and institutionalizing police-public health collaboration; ^E^ The rate of HIV transmission and number of PLHIV were estimated based on national surveillance data; ^F^ Study included a comparison population drawn from cross-sectional data collected in areas where the policy/intervention was not being implemented; ^G^ PLHIV = people living with HIV; ^H^ Vulnerable groups referenced: women, young people, MSM, sex workers, PWID, prisoners and migrants

### Quality assessment

Two reviewers (DS and KS) assessed the quality of the selected articles (Table 2). For quantitative articles, quality was assessed using a modified Downs and Black checklist with 19 items covering four sub-scales: reporting, external validity, bias, and confounding [47]. Eight items (9, 14, 15, 17, 19, 21, 26 and 27) are relevant only to trials and cohort studies and were removed from the checklist.

**Table 2:** Quality of the 23 studies reviewed

1st author, publication date	Study Design^a^	Summary Score for Quality Critique
*Quantitative (Modified Downs and Black, 1998)*		
Beletsky, 2011 [59]	RXS	73.7% (14/19)
Beletsky, 2013 [65]	XS	63.2% (12/19)
Beletsky, 2012 [66]	XS	63.2% (12/19)
Du, 2011 [42]	XS	73.7% (14/19)
Ellen, 2015 [60]	RXS	68.4% (13/19)
Fang, 2004 [64]	RXS	73.7% (14/19)
Grangeiro, 2011 [67]	RXS^B^	73.7% (14/19)
Homaifar, 2005 [56]	XS	26.3% (5/19)
Martinez, 2007 [61]	RXS	57.9% (11/19)
Rich, 2007 [62]	RXS^B^	63.2% (12/19)
Sarnquist, 2007 [43]	RXS	57.9% (11/19)
van Rensburg, 2007 [58]	XS	63.2% (12/19)
*Qualitative (Spencer* et al. *2003)*		
Argento, 2011 [63]	QP	88.9% (16/18)
Dworkin, 2014 [55]	QP	77.8% (14/18)
Jones, 2005 [57]	QP	61.1% (11/18)
*Mixed Methods (Spencer* et al. *2003)*		
Gruskin, 2013 [25]	QP^C^	88.9% (16/18)
Jardine, 2012 [49]	QP^C^	66.7% (12/18)
*Other*		
Ahmad, 2013 [51]	PR	n/a
Ainsworth, 2003 [52]	PR	n/a
Gruskin, 2009 [53]	PR	n/a
Gurnani, 2011 [54]	PMD	n/a
Jeffreys, 2011 [50]	PR	n/a
Lazariu, 2015 [44]	PMD	n/a

^a^Study design abbreviations: XS = observational cross-section RXS = observational repeated cross-sections; QP = qualitative post-test only; MM = mixed methods; PR = policy review; PMD = program monitoring data; ^B^ These studies included a comparison population drawn from cross-sectional data collected in areas where the policy/intervention was not being implemented; ^C^ The quantitative data included in these studies was drawn from document reviews and/or program monitoring data, we therefore decided to assess their quality based on the qualitative assessment tool; N/a = these studies could not be scored using either method as they either relied solely on monitoring data to evaluate the intervention, or were policy reviews, which analysed data from multiple previously published studies

The maximum score for the modified checklist was 19. While the Downs and Black checklist does not have a pre-determined cut-off point to assess the quality of a paper, for this review, studies scoring between 0 and 6 were considered ‘poor quality’, studies scoring between 7 and 12 were considered ‘fair quality’, and studies scoring between 13 and 19 were considered ‘good quality’.

The Spencer guide for critically appraising qualitative research was used to assess the qualitative studies [48]. Quality was evaluated using 18 items comprising 9 sub-scales: findings, design, sample, data collection, analysis, reporting, reflexivity and neutrality, ethics and auditability [48]. Scores were interpreted as follows: 0–5 ‘poor quality’, 6–12 ‘fair quality’, and 13–18 ‘good quality. We also used the Spencer guide to assess the quality of the two mixed-methods studies [25, 49]. We were unable to assess the quality of six articles [44, 50–54], since they were either policy reviews or utilized program monitoring data only.

### Data synthesis

Due to the lack of uniform reporting of primary and secondary outcomes across the 23 articles, we did not conduct a meta-analysis. Instead, studies were categorized on the UNAIDS’ human rights program categories, whether advancing human rights was implicitly or explicitly mentioned in the intervention description, if HRBA principles were incorporated, if the interventions involved the expansion of the right to health attributes for the intended recipients, and the socio-ecological level(s) addressed (See Additional file 1: S1).

## Results

**Fig. 1:**
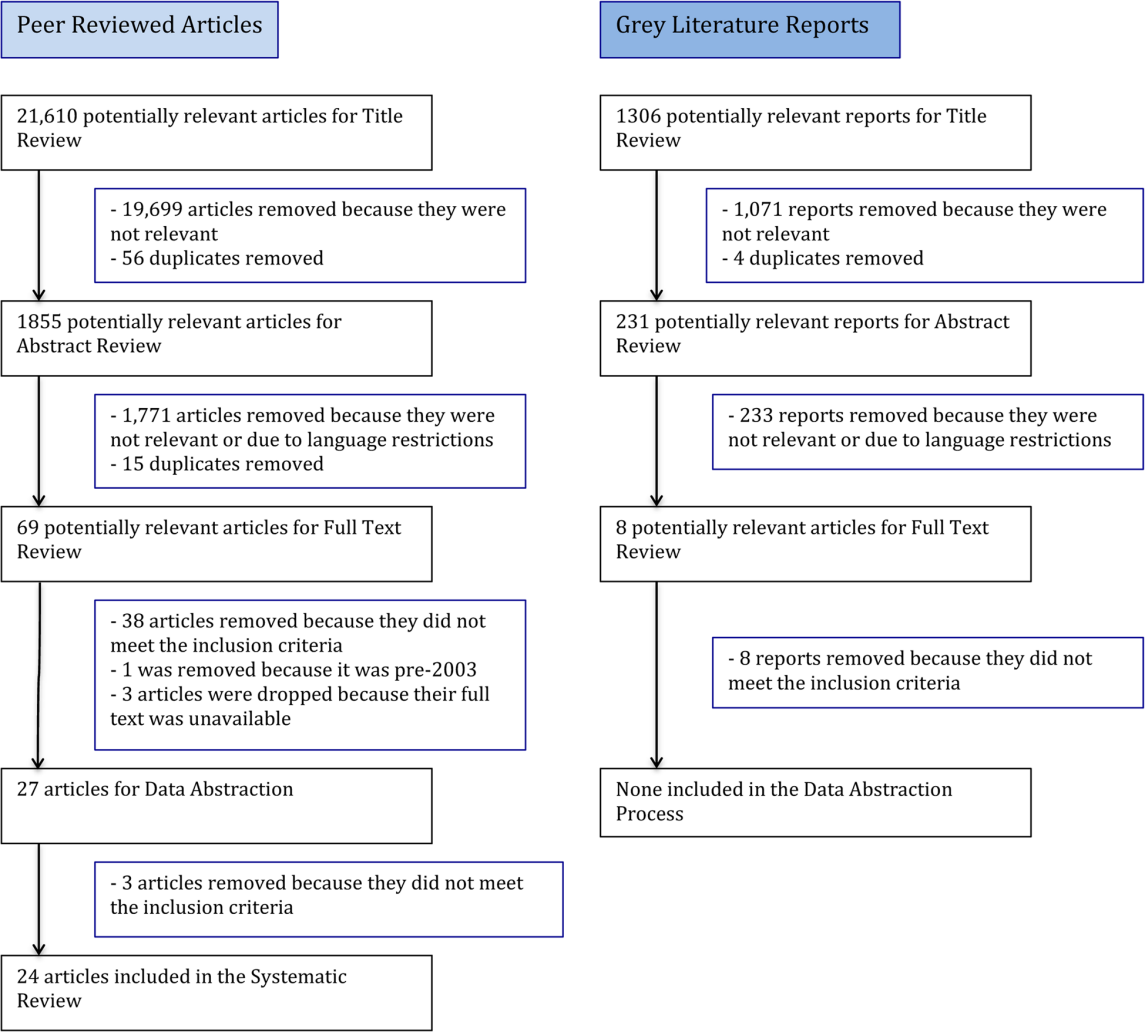
Flow chart of search strategy

The search criteria identified 31,861 potentially relevant articles and reports. After removing 10,251 duplicates, 21,610 peer-reviewed articles and 1306 grey literature reports were included in the title review phase (Fig. 1). A total of 23 peer-reviewed articles met the inclusion criteria and were included for further analysis.

### Study and intervention characteristics

The studies spanned a large geographical area. Six studies were conducted in East and Southern Africa [25, 51, 55–58] and seven were conducted in North America [42–44, 59–62]. Six studies were conducted in the Asia and Pacific region [49, 50, 52, 54, 63, 64] and two were conducted in Eastern and Central Asia [65, 66]. One study was conducted in Latin America [67], one in the Caribbean [60] and another, by Gruskin et al. was a multi-country study examining data from 133 countries [53]. No studies from Western and Central Europe, West and Central Africa or the Middle East and North Africa were identified. The most represented countries were the United States (7 studies), South Africa (3 studies), Kyrgyzstan (2 studies), Kenya (2 studies) and India (2 studies) (Table 1).

The interventions focused on a wide variety of populations. Sixteen addressed a single population, the most common of which were reproductive aged adults [42, 44, 64, 67], police [49, 59, 65, 66], sex workers [50, 52, 56], and people who inject drugs (PWID) [61, 62]. Other populations addressed included at-risk youth [60], pregnant women [43] and women [55]. Eight interventions focused on multiple populations [25, 51, 53, 54, 57, 58, 63] (Table 1).

**Fig. 2:**
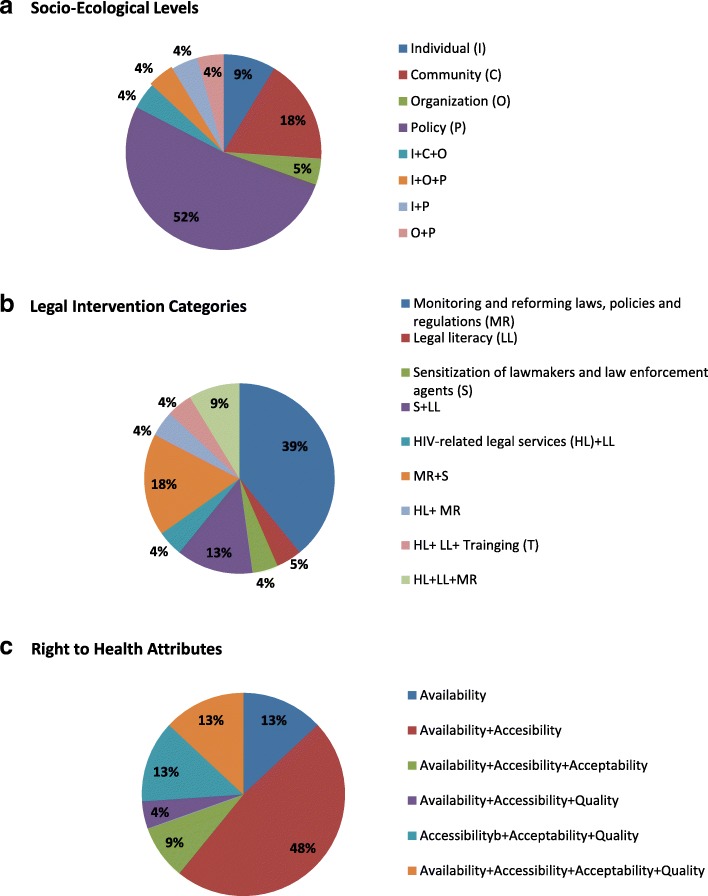
Socio-ecological levels, legal intervention categories and right to health attributes of 23 studies reviewed

Almost half of the interventions evaluated addressed a single UNAIDS’ program category. Nine interventions fell within the monitoring and reforming laws, policies and regulations category [42–44, 56, 57, 61, 62, 64, 67], one study examined a legal literacy intervention [58], and one study evaluated an intervention to sensitize law enforcement agents [63]. Nine studies addressed two intervention categories. The most common combination was monitoring and reforming laws and sensitization of lawmakers [49, 50, 52, 60]. Legal literacy and sensitization of law makers were combined in three studies [59, 65, 66]. One study addressed both HIV-related legal services and legal literacy [55] and one addressed HIV-related legal services and monitoring and reforming laws [53]. Two studies addressed three of the UNAIDS’ program areas [25, 51]. Lastly, one intervention in India addressed four program areas, including: HIV-related legal services, monitoring and reforming laws and policies, legal literacy and sensitization of law enforcement agents [54] (Fig. 2a, Table 1).

Seventeen studies (74%) intervened at a single socio-ecological level. Public policy interventions were the most common (12 studies), followed by community (4 studies), individual (2 studies) and organizational (1 study). Four studies addressed multiple-levels [51, 54, 56, 60] (Fig. 2b; Table 1). Of the 23 studies included in the review, only five (22%) explicitly referenced the promotion and protection of human rights in the description of the intervention [25, 51, 53, 54, 57]. Most interventions (74%) incorporated two or more HRBA principles in either design or intent. Eleven interventions incorporated two principles [25, 49, 50, 53–55, 58, 60, 62, 63, 67], three incorporated three principles [51, 61, 66], and three incorporated all five principles [51, 52, 65]. The combinations of principles incorporated varied widely (Table 1).

Twenty studies (87%) evaluated interventions that incorporated or sought to expand two or more attributes of the right to health. Eleven interventions sought to influence both availability and accessibility [42, 43, 50–53, 56–58, 60, 61]. Six interventions incorporated availability, accessibility, and acceptability [55, 59, 63–66] and three studies sought to influence all four elements of the right to health [25, 54, 67]. The three interventions that incorporated only one element all sought to influence availability [44, 49, 62] (Fig. 2c, Table 1).

### Study design

None of the studies employed an experimental design. All of the quantitative studies were observational, with seven employing repeated cross-sectional surveys [43, 59–62, 64, 67] and five employing a single cross-sectional survey [42, 56, 58, 65, 66]. Two studies employed mixed methods [25, 49] and three collected qualitative data following the intervention [55, 57, 63]. Two studies analyzed program monitoring data [44, 54] and four studies were policy reviews that utilized analytic frameworks to evaluate a policy or law based on a review of previously published data [50–53] (Table 1).

### Study duration and outcomes

Due to the diversity of interventions, the intervention duration and outcomes varied considerably. Among studies assessing the impact of a law, policy, or programme, some, like the legalization of sex work in Senegal [56], came into effect as early as 1969 and others, like the law in New York State mandating the offer of opt-out, provider-initiated HIV testing [44], as late as 2010. In the cases where studies assessed interventions involving a targeted training or course, the duration ranged from one, 30-min session to a 46-h, multi-session training [59, 65].

The majority (83%) of studies reported improvements in HIV-related health outcomes assessed. With regards to human rights-related barriers to HIV services, the police training programs evaluated noted increased knowledge and attitudes about harm reduction and increased knowledge of laws and policies related to detention of key populations [59, 65, 66]. Likewise, a sex worker-led HIV prevention program in India that engaged police and lodge owners reported reduced experiences of violence from police [63]. One intervention in Kenya positively influenced knowledge of human rights among both rights holders (PLHIV) and duty bearers, including health care workers facilitating access to legal aid to claim rights for PLHIV [25].

Three interventions either had no influence on the human rights-related barrier to HIV services examined (i.e. illegal policing practices, lack of knowledge of harm reduction, etc.) or had a negative influence. In one case, a national policy on harm reduction in Vietnam did not influence the way in which police engaged with PWID at the ward level [49]. Similarly, protective national laws in South Africa did not reduce human rights violations among PLHIV in two communities studied by Jones et al. [57]. In California, the legalization of syringe exchange programs (SEPs) in some counties led to an increase in arrests of PWID and citations for drug paraphernalia [61]. Failure of these interventions to bring about change appears to be related to incomplete efforts or limited enforcement and dissemination efforts.

Twelve studies reported positive influences on HIV prevention, care and treatment outcomes [42–44, 50–52, 56, 58, 60, 62, 64, 67]. The interventions evaluated ranged from policies that mandated the offering of opt-out, provider-initiated HIV testing among reproductive aged adults and pregnant women in New York and California, respectively [42, 43], to legalization of syringe sales at pharmacies in Rhode Island [62], to the implementation of a structured response to HIV in Brazil [67]. Two policy reviews of the national response to HIV in Thailand and China, found positive outcomes of the 100% condom use policy for increasing condom use among sex workers [50, 52]. One study by Ellen et al. found no effect of structural changes implemented at organizational and policy-levels on condom use or HIV testing among at-risk youth in the U.S. and Puerto Rico [60].

The implementation of free access to antiretroviral treatment in Taiwan in 1997 led to a 53% decrease in the rate of HIV transmission and no change in the incidence of syphilis among reproductive aged adults [64]. Likewise, the activities conducted by the Treatment Action Campaign in South Africa, namely strategic litigation, led to free access to ARV drugs, which in turn increased access to treatment for PLHIV and reductions in HIV transmission as a result [51]. The study evaluating Thailand’s response to HIV cited the human rights-based approach adopted in the government’s response to AIDS as key to the reduction observed in HIV incidence [52] (Table 1).

### Quality assessment

Eleven studies employed exclusively quantitative methods and one mixed methods study predominately employed quantitative methods. These twelve studies were assessed with the modified Downs and Black checklist [47]. Three studies employed exclusively qualitative methods and two mixed methods studies predominately employed qualitative methods. These five studies were assessed based on the Spencer et al. checklist [48]. Seven of the studies included in this review employed a study design centered on policy review or program monitoring. As these study designs do not fit the criteria for review using the Downs and Black and the Spencer et al. checklists, and there is no other appropriate assessment tool that can be applied, we were not able to assess the quality of these studies.

Overall, we found most studies to be of fair quality with six studies (three qualitative and three quantitative) scoring in the good-quality range (Table 2).

## Discussion

This systematic review revealed promising evidence of the impact of human rights programs on HIV-related outcomes for people living with HIV and key and vulnerable populations most at risk of HIV, ranging from decreased HIV risk behaviors to increased HIV testing to reduced incidence. Human rights programs to improve HIV-related health outcomes have evolved and grown in practice since the UN adopted the common understanding of a human rights-based approach in 2003, with a diversity of approaches being employed. Yet evaluation efforts have not kept pace, leaving critical questions for implementation and scale-up of these efforts at local, state and national-levels.

The studies spanned a large geographical area and were typically complex in nature. While interventions were completed across country income levels, low and middle-income countries had fewer total studies per country. In addition, there was limited evidence published from Western and Central Europe, West and Central Africa and the Middle East and North Africa, which is disappointing given the range of legal and human rights contexts across these settings. Overall, the studies that showed a positive influence on HIV-related health outcomes were of fair to good quality. While most interventions addressed a single population, the populations were diverse, ranging from duty bearers, such as police and health care workers, to rights holder, including PLHIV and pregnant women.

All five socio-ecological levels of influence were addressed across the 23 interventions assessed, sometimes in combination, yet most interventions focused on a single socio-ecological level, namely public policy. This finding highlights the importance of enabling legal and policy environments for structural interventions aimed at respecting, protecting and promoting human rights. The majority of interventions addressed 2 or more of the 5 UNAIDS’ human rights programs [16]. Monitoring and reforming laws, policies and regulations and sensitizing lawmakers & law enforcement agents were the most commonly combined. These findings are encouraging, as they reflect the complexity required to shift the structures and norms that hinder access to HIV services among marginalized and key populations [19]. Moving forward, the field would benefit considerably from the development of an overarching research framework, and accompanying indicators, that capture the complexity of human rights programs and reflect implementation realities on the ground.

Most interventions sought to influence two or more attributes of the right to health, namely availability and accessibility. The focus on accessibility in so many of the studies [25, 42, 43, 50–61, 63–67] included in our review marks an important step forward in the field. While availability of HIV services has increased over the last decade [8–10], we have fallen short at increasing access to health among marginalized and key populations [7, 13, 14].

Despite these positive findings, we identified several challenges that need to be addressed in future research on human rights programs to enhance HIV outcomes. A limited number of studies explicitly referenced the promotion and protection of human rights in the description of the intervention evaluated [25, 51, 52, 54, 57]. Given the broad ratification of core human rights treaties by member-states, the UN’s clear articulation of the human rights-based approach [26], and the subsequent adaptation for the health context [25], researchers should be more clear in describing how the HRBA was applied at all stages of intervention development and implementation. This clarity would allow a more nuanced understanding of the HRBA, including identification of areas requiring further attention and support.

Of concern, some of the interventions assessed seemed not to consider human rights implications at all. For example, three studies assessed US state laws that mandated the offering provider-initiated, opt-out testing for reproductive aged adults [42, 44] and pregnant women [43]. All three studies found significant increases in testing following the passage of the law. Yet, no effort was made to capture whether due diligence occurred aiming to get informed consent prior to the testing, or the potentially harmful effects of these laws for people who learned their HIV status through provider-initiated testing. Likewise, the 100% condom use policy, in the absence of meaningful participation of sex workers in its design and implementation, can perpetuate vulnerability to abuse and exploitation.

In addition, we found that laws intended to protect key populations, if incomplete or not accompanied by proper enforcement, can be harmful or ineffective. Such was the case in California, where arrests of PWID increased in counties that legalized syringe-exchange programs compared with those who had not, as PWID became more visible in the community and easier for police to identify while carrying multiple syringes, a punishable offense [61]. This study also highlights the need for comprehensive legislation that addresses all of the issues impeding harm reduction. For example, if the legalization of SEPs was coupled with the legalization of carrying more than one syringe, the intervention may have been more effective at enabling harm reduction services [68]. Overall, there is a need for human rights programs to go further, moving beyond changing laws and policies to support proper enforcement of such changes.

Similarly, in Vietnam [49] and South Africa [57] the introduction of supportive legal environments to protect key populations seem to have failed due to a lack of awareness of the laws, which impeded implementation at the community-level. These findings suggest that those who engage with key populations directly, including police and health care workers, need training so they are aware of the current laws and policies. This knowledge will enable them to ensure that legal protections and procedures are implemented to minimize harm and prevent HIV transmission. These findings also suggest the importance of interventions aimed at increasing knowledge of rights and relevant legal environment by the rights-holders themselves, as well as the importance of access to justice, remedies and redress where rights are violated [69, 70].

To minimize harm and protect the rights of PLHIV and key populations, duty bearers (i.e. researchers, politicians, health care providers, etc.) need to actively engage members of key and affected populations during intervention, policy and law development, implementation, and monitoring and evaluation. In addition, evaluation of the impact of human rights programs, laws and policies should be expanded beyond HIV-related outcomes alone, to include their role in respecting, protecting and promoting human rights, as well as their potential harms.

Lastly, focusing on changing structures without also addressing individuals is another potential barrier to the success of human rights programs. For example, a multi-state study of locally identified structural changes within institutions, like homeless shelters and schools, reported no significant effect on reductions of sexual risk behaviour among at-risk teens in the US [60]. These findings suggest that structural interventions may work best when combined with other types of interventions, like behavioural and biomedical, and when they address multiple socio-ecological levels.

### Limitations

There are limitations to the approach utilized here. Some human rights program areas were underrepresented in our review, including strategic litigation, namely due to methodological challenges evaluating such approaches. Given the importance of strategic litigation for setting legal precedent [71], this approach should not be discounted in a comprehensive HIV response. Legal interventions often apply to entire populations (i.e. national laws), which precludes a control group. Other legal interventions are part of a structural approach with multiple components occurring at multiple levels, and thus are not conducive to the classic RCT design. Additional research and the development of alternative or new evaluation methodologies, such as propensity scores, difference-in-difference designs, causal inference, and structural equation modelling, are needed to strengthen the rigor of the evidence on human rights programs. These approaches may provide additional insight in leveraging cross-sectional data to understand causal relationships and potentially even characterize pathways of action between human rights programs and ultimately, health outcomes. Some studies of human rights programs may have been missed by restricting inclusion to articles published in English. Future studies should include studies published in additional languages.

Most of the selected papers did not explicitly incorporate the principles of a human rights-based approach or the attributes of the Right to Health (see Additional file 1: S1). Consequently, inferences were made from the data and results to determine the attention paid to each. Given the nature of this field and the kinds of interventions evaluated, a limited number of studies analyzed data collected prior to and following the intervention. For this reason, our inclusion criteria allowed for cross-sectional surveys and the use of national surveillance data for analysis.

A meta-analysis was not completed due to the diverse nature of the interventions and outcomes, thereby limiting the assessment of the effectiveness of pooled interventions at demonstrating the link between human rights programs and HIV-related outcomes. Generalizability of the findings was also limited because of the small sample sizes and specific population focus of many of the interventions. This also impeded the assessment of causality, as none of the studies included randomized control groups. The HIV-related outcomes assessed varied widely, making it difficult to compare across the interventions studied. Despite these limitations, and our specific inclusion criteria, this review assessed 23 wide-ranging studies, representing numerous populations, interventions and study locations, thereby giving it strength and value.

## Conclusions

Our review is the first to systematically examine the evidence on the impact of human rights programs on HIV-related outcomes. Our findings suggest great promise for human rights programs as part of comprehensive responses to HIV at the local, state and national levels, yet more evidence is needed to guide implementation and scale-up. Investments in the implementation and evaluation of human rights programs have been minimal to date [72]. The resulting lack of evidence is hampering the scale-up of interventions to foster the supportive legal, social, political and economic environments needed to reach and engage in care key and vulnerable populations most at risk of HIV infection. Now is the time to ensure that human rights are front and center in a comprehensive HIV response by increasing investment in human rights programs and in their rigorous evaluation.

## Additional files

Additional file 1: **S1.** Details of Categorization Principles. (DOCX 121 kb)

Additional file 2: **S2.** Search strategy. (DOCX 28 kb)

Additional file 3: **S3.** Standardized data abstraction form. (DOCX 17 kb)

## Abbreviations

AIDS: Acquired Immunodeficiency Syndrome
ARV: Antiretroviral Drug
HIV: Human Immunodeficiency Virus
HRBA: Human Rights-Based Approach
LGBTQ: Lesbian, Gay, Bisexual, Transgender, and Questioning
MM: Mixed Methods
MSM: Men who have Sex with Men
PAIS: Public Affair Information Service
PLHIV: People Living with HIV
PMD: Program Monitoring Data
PR: Policy Review
PRISMA: Preferred Reporting Items for Systematic Reviews and Meta-Analyses
PWID: Persons who Inject Drugs
QP: Qualitative Post-Test Only
RCT: Randomized Control Trial
RXS: Observed Repeated Cross-sections
SEP: Syringe Exchange Program
UN: United Nations
UNAIDS: The Joint United Nations Programme on HIV/AIDS
UNESCO: United Nations Education, Scientific and Cultural Organization
USAID: The United States Agency for International Development
WHO: World Health Organization
XS: Observational Cross-section
