# Supplementary material for: A systematic review of selected human rights programs to improve HIV-related outcomes from 2003 to 2015: what do we know?
Source: BMC Infect Dis. 2019 Mar 5;19:209. doi: 10.1186/s12879-019-3692-1 (PMC6399958; doi:10.1186/s12879-019-3692-1)
Supplement: Supplementary file 1 — S1. Details of Categorization Principles. (DOCX 121 kb) [file 12879_2019_3692_MOESM1_ESM.docx]

S1. Details of Categorization Principles.

| **UNAIDS’ Categories of Human Rights Programs [16]** | |
| --- | --- |
| HIV-related legal services | Legal information and referrals; Legal advice and representation; Alternative/community forms of dispute resolution; Engaging religious or traditional leaders and traditional legal systems (e.g. village courts) with a view to resolving disputes and changing harmful traditional norms; and Strategic litigation. |
| Monitoring and reforming laws, regulations and policies relating to HIV | Review of laws and law enforcement practices to see whether they impact the response to HIV positively or negatively; Assessment of access to justice for people living with or vulnerable to HIV; Advocacy and lobbying for law reform; Engagement of Parliamentarians and Ministers, religious and traditional leaders, among others; and Promotion of the enactment and implementation of laws, regulations and guidelines that prohibit discrimination and support access to HIV prevention, treatment, care and support. |
| Legal literacy | Awareness-raising campaigns that provide information about rights and laws related to HIV through media (e.g. TV, radio, print, Internet); Community mobilization and education; Peer outreach; and Telephone hotlines. |
| Sensitization of law-makers and law enforcement agents | Sensitization of police regarding HIV and how it is and is not transmitted; the importance of reaching out to and accessing populations at risk; the importance of appropriately addressing domestic and sexual violence cases in the context of HIV; and the negative consequences of illegal police activity on justice and on the HIV response; Information and sensitization sessions for Parliamentarians, personnel of Ministries of Justice and Interior, judges, prosecutors, lawyers, and traditional and religious leaders on the legal, health and human rights aspects of HIV and on relevant national laws and the implications for enforcement, investigations and court proceedings; and HIV in the Workplace program for law makers and enforcers. |
| Training on human rights and medical ethics | Human rights and ethics training conducted with: Individual health care providers, Health care administrators, and Health care regulators. |
| **Attributes of the Right to Health [27]** | |
| Availability | A sufficient quantity of functioning public health and health care facilities, goods and services, as well as programs. |
| Accessibility | Health facilities, goods and services should be accessible to everyone. Accessibility has four overlapping dimensions:  a. non-discrimination; b. physical accessibility; c. economical accessibility (affordability); d. information accessibility. |
| Acceptability | All health facilities, goods and services must be respectful of medical ethics and culturally appropriate as well as sensitive to gender and life-cycle requirements. |
| Quality | Health facilities, goods and services must be scientifically and medically appropriate and of good quality. |
| **Principles of a Human Rights-Based Approach [25;26;74]** | |
| Empowerment | Ensure that rights-holders and duty-bearers share a common understanding of human rights goals; and must also ensure that systems are in place to educate and raise awareness of all relevant stakeholders. |
| Non Discrimination | Recognize that some groups are more vulnerable than others; and pay special attention to their needs. |
| Participation | Ensuring that all those who will be affected by the intervention have an opportunity to have their say and contribute to it. |
| Accountability | Ensure that there is proper accountability for meeting these principles. |
| Linkages | Ensuring that in planning, policy and service delivery there is an analysis of which human rights are relevant, who the rights holders are, and who is responsible for ensuring that those rights are protected, promoted and fulfilled. |
