# Supplementary material for: A systematic review of selected human rights programs to improve HIV-related outcomes from 2003 to 2015: what do we know?
Source: BMC Infect Dis. 2019 Mar 5;19:209. doi: 10.1186/s12879-019-3692-1 (PMC6399958; doi:10.1186/s12879-019-3692-1)
Supplement: Supplementary file 2 — S2. Search strategy. (DOCX 28 kb) [file 12879_2019_3692_MOESM2_ESM.docx]

**Table S2. Search Straegy**

1. **Peer reviewed**
   1. **PubMed: n=4710**
2. **Legal:** "Health Policy"[Mesh] OR health polic*[tw] OR "Legislation as Topic"[Mesh] OR "Healthcare Disparities"[Mesh] OR legal*[tw] OR law*[tw] OR paralegal*[tw] OR rights[tw] OR structural intervention*[tw]
3. **Population:** "Vulnerable Populations"[Mesh] OR vulnerable group*[tw] OR vulnerable population*[tw] OR marginali*[tw] OR HIV*[tw] OR key population*[tw] OR "HIV Infections"[Mesh]
4. **Evaluation:** "Health Plan Implementation/methods"[MeSH] OR "Epidemiologic Methods"[MeSH] OR "Qualitative Research"[MeSH] OR quantitative*[tw] OR qualitative*[tw] OR evidence[tw] OR analy*[tw] OR intervention*[tw] OR "Intervention Studies"[Mesh] OR evaluat*[tw] OR training*[tw] OR monitor*[tw] OR assess*[tw] OR impact*[tw] OR implement*[tw]
   1. **Scopus: N=10572** in Scopus, it is necessary to limit the search to the subject area of interest—here, Health Sciences and Social Sciences & Humanities, excluding non-relevant subject areas (i.e., veterinary science, material sciences, &c). Note: This search is not picking up Beletsky 2013.

Search fields: (TITLE-ABS-KEY)

Exclusionary terms:

ND  DOCTYPE ( **ar**  OR **re** )  AND  SUBJAREA  ( **mult**  OR  **medi**  OR  **nurs**  OR  **vete**  OR  **dent**  OR  **heal**  OR  **mult**  OR  **arts**  OR  **busi**  OR  **deci**  OR  **econ**  OR  **psyc**  OR  **soci** )  AND  PUBYEAR  >  **2005**  AND  ( EXCLUDE ( SUBJAREA ,  **"IMMU"** )  OR  EXCLUDE ( SUBJAREA ,  **"PHAR"** )  OR  EXCLUDE ( SUBJAREA ,  **"BIOC"** )  OR EXCLUDE ( SUBJAREA ,  **"VETE"** ) )  AND  ( EXCLUDE ( SUBJAREA ,  **"MATH"** )  OR  EXCLUDE ( SUBJAREA ,  **"ENVI"** )  OR  EXCLUDE ( SUBJAREA ,  **"AGRI"** )  OR  EXCLUDE ( SUBJAREA ,  **"ENGI"** )  OR  EXCLUDE ( SUBJAREA , **"COMP"** )  OR  EXCLUDE ( SUBJAREA ,  **"BUSI"** )  OR  EXCLUDE ( SUBJAREA ,  **"PHYS"** )  OR  EXCLUDE ( SUBJAREA ,  **"CENG"** )  OR  EXCLUDE ( SUBJAREA ,  **"CHEM"** )  OR  EXCLUDE ( SUBJAREA ,  **"MATE"** ) )

1. **Legal:** “health polic*” OR legal* OR law* OR paralegal OR rights OR “structural intervention*”

**(B) Population: “**vulnerable group*” OR “vulnerable population*” OR marginali* OR HIV* OR “key population*”

**(C) Evaluation:** quantitative* OR qualitative* OR evidence OR analy* OR intervention* OR evaluat* OR training* OR monitor* OR assess* OR impact* OR implement*

- 1. **Embase: 10676** [article]/lim AND [humans]/lim AND [articles]/lim OR [review]/im OR [article in press]/lim AND [embase]/lim AND [2003-2015]/py

1. **Legal:** (health NEXT/1 polic*):de,cl,ab,ti OR legal*:de,cl,ab,ti OR law*:de,cl,ab,ti OR paralegal*:de,cl,ab,ti OR rights:de,cl,ab,ti OR (structural NEXT/3 intervention*):de,cl,ab,ti
2. **Population:** (vulnerable NEXT/3 group*):de,ab,ti OR (vulnerable NEXT/3 population*):de,ab,ti OR marginali*:de,ab,ti OR hiv*:de,ab,ti OR (key NEXT/1 population*):de,ab,ti
3. **Evaluation:** quantitative*:de,cl,ab,ti OR qualitative*:de,cl,ab,ti OR evidence:de,cl,ab,ti OR analy*:de,cl,ab,ti OR intervention*:de,cl,ab,ti OR evaluat*:de,cl,ab,ti OR training*:de,cl,ab,ti OR monitor*:de,cl,ab,ti OR assess*:de,cl,ab,ti OR impact*:de,cl,ab,ti OR implement*:de,cl,ab,ti
   1. **POPLINE: 1055** This search returns only Beletsky 2013; the others are not indexed by this database.
4. **Legal:** “health polic*” OR legal* OR law* OR paralegal OR rights OR “structural intervention*”
5. **Population: “**vulnerable group*” OR “vulnerability u population*” OR marginali* OR HIV* OR “key population*”

**(C) Evaluation:** quantitative* OR qualitative* OR evidence OR analy* OR intervention* OR evaluat* OR training* OR monitor* OR assess* OR impact* OR implement*

- 1. **PAIS International: N=890**. This search returns only Abdikeeva 2013; the others are not indexed by this database.

1. **Legal:** ALL(health PRE/1 polic* OR legal* OR law* OR paralegal OR rights OR structural PRE/1 intervention*)
2. **Population:** ALL(vulnerable PRE/3 group* OR vulnerable PRE/3 population* OR marginali* OR HIV* OR key PRE/1 population*)
3. **Evaluation:** ALL(quantitative* OR qualitative* OR evidence OR analy* OR intervention* OR evaluat* OR training* OR monitor* OR assess* OR impact* OR implement*)
   1. **Sociological Abstracts (formerly Sociofile): N=1219**
4. **Legal:** ALL(health PRE/1 polic* OR legal* OR law* OR paralegal OR rights OR structural PRE/1 intervention*)
5. **Population:** ALL(vulnerable PRE/3 group* OR vulnerable PRE/3 population* OR marginali* OR HIV* OR key PRE/1 population*)
6. **Evaluation:** ALL(quantitative* OR qualitative* OR evidence OR analy* OR intervention* OR evaluat* OR training* OR monitor* OR assess* OR impact* OR implement*)
   1. **Global Health (OVID): N=2776**
7. **Legal:** ((health adj1 polic*) or legal* or law* or paralegal* or rights* or (structural adj1 intervention*)).mp. or exp law/ or exp legal rights/ or exp human rights/
8. **Population:** ((vulnerable adj1 group*) or (vulnerable adj1 population*) or marginali* or HIV* or (key adj1 population*)).mp. or exp HIV infections/ [mp=abstract, title, original title, broad terms, heading words, identifiers, cabicodes]
9. **Evaluation:** (quantitative* or qualitative* or evidence or analy* or intervention* or evaluat* or training* or monitor* or assess* or impact* or implement*).mp. [mp=abstract, title, original title, broad terms, heading words, identifiers, cabicodes]
10. **Non-Peer Reviewed**
    1. **Scopus: N=843**
11. **Legal:** TITLE-ABS-KEY("health polic*" OR legal* OR law* OR paralegal OR rights OR "structural intervention*"))
12. **Population:** TITLE-ABS-KEY("vulnerable group*" OR "vulnerable population*" OR marginali* OR HIV* OR "key population*")
13. **Evaluation:** (TITLE-ABS-KEY(quantitative* OR qualitative* OR evidence OR analy* OR intervention* OR evaluat* OR training* OR monitor* OR assess* OR impact* OR implement*)

**Limiting:**

SUBJAREA(MULT OR MEDI OR NURS OR VETE OR DENT OR HEAL OR MULT OR ARTS OR BUSI OR DECI OR ECON OR PSYC OR SOCI) AND PUBYEAR > 2002 AND ( LIMIT-TO(SUBJAREA,"IMMU" ) OR LIMIT-TO(SUBJAREA,"PHAR" ) OR LIMIT-TO(SUBJAREA,"BIOC" ) OR LIMIT-TO(SUBJAREA,"ARTS" ) OR LIMIT-TO(SUBJAREA,"ECON" ) OR LIMIT-TO(SUBJAREA,"NEUR" ) OR LIMIT-TO(SUBJAREA,"MEDI" ) OR LIMIT-TO(SUBJAREA,"SOCI" ) ) AND ( EXCLUDE(SUBJAREA,"IMMU" ) OR EXCLUDE(SUBJAREA,"PHAR" ) OR EXCLUDE(SUBJAREA,"BIOC" ) OR EXCLUDE(SUBJAREA,"ARTS" ) OR EXCLUDE(SUBJAREA,"ECON" ) OR EXCLUDE(SUBJAREA,"NURS" ) OR EXCLUDE(SUBJAREA,"NEUR" ) OR EXCLUDE(SUBJAREA,"HEAL" ) OR EXCLUDE(SUBJAREA,"VETE" ) OR EXCLUDE(SUBJAREA,"AGRI" ) OR EXCLUDE(SUBJAREA,"ENVI" ) OR EXCLUDE(SUBJAREA,"BUSI" ) OR EXCLUDE(SUBJAREA,"DENT" ) OR EXCLUDE(SUBJAREA,"MATH" ) OR EXCLUDE(SUBJAREA,"ENGI" ) OR EXCLUDE(SUBJAREA,"COMP" ) OR EXCLUDE(SUBJAREA,"DECI" ) OR EXCLUDE(SUBJAREA,"EART" ) OR EXCLUDE(SUBJAREA,"PHYS" ) OR EXCLUDE(SUBJAREA,"CHEM" ) OR EXCLUDE(SUBJAREA,"CENG" ) OR EXCLUDE(SUBJAREA,"MATE" ) OR EXCLUDE(SUBJAREA,"ENER" ) OR EXCLUDE(SUBJAREA,"MULT" ) ) AND ( EXCLUDE(DOCTYPE,"ar" ) OR EXCLUDE(DOCTYPE,"re" ) ) AND ( EXCLUDE(DOCTYPE,"ip" ) ) AND ( EXCLUDE(SRCTYPE,"j" ) )

- 1. **Popline: N=484**

1. **Legal:** “health polic*” OR legal* OR law* OR paralegal OR rights OR “structural intervention*”
2. **Population: “**vulnerable group*” OR “vulnerability u population*” OR marginali* OR HIV* OR “key population*”

**(C) Evaluation:** quantitative* OR qualitative* OR evidence OR analy* OR intervention* OR evaluat* OR training* OR monitor* OR assess* OR impact* OR implement*

- 1. **PAIS International: N=5**

1. **Legal:** (ALL(health PRE/1 polic* OR legal* OR law* OR paralegal OR rights OR structural PRE/1 intervention*)
2. **Population:** ALL(vulnerable PRE/3 group* OR vulnerable PRE/3 population* OR marginali* OR HIV* OR key PRE/1 population*)
3. **Evaluation:** ALL(quantitative* OR qualitative* OR evidence OR analy* OR intervention* OR evaluat* OR training* OR monitor* OR assess* OR impact* OR implement*))

Limiting: AND at.exact("Article") AND at.exact("Government & Official Document" OR "News")
