# Supplementary material for: A systematic review of selected human rights programs to improve HIV-related outcomes from 2003 to 2015: what do we know?
Source: BMC Infect Dis. 2019 Mar 5;19:209. doi: 10.1186/s12879-019-3692-1 (PMC6399958; doi:10.1186/s12879-019-3692-1)
Supplement: Supplementary file 3 — S3. Standardized data abstraction form. (DOCX 17 kb) [file 12879_2019_3692_MOESM3_ESM.docx]

**S3. Standardized Data Abstraction Criteria**

1. Author
2. Title
3. Year
4. Reviewer
5. Location
6. Region
7. Intervention level
   1. Individual
   2. Interpersonal
   3. Organizational
   4. Community
   5. Public policy
   6. Multiple
8. Target population
   1. Law makers
   2. Police
   3. Health care workers
   4. Sex workers
   5. People who inject drugs
   6. Pregnant women
   7. Reproductive aged adults
   8. People living with HIV (PLHIV)
   9. Survivors of gender-based violence (GBV)
   10. Women
   11. LGBTQ
   12. At-risk youth
   13. Multiple
9. Details of ‘multiple’ target populations (e.g. which ones specifically)
10. Sample
11. Study design
    1. Quantitative post-test only
    2. Pre/post-test with a control group
    3. Pre/post test with no control group
    4. Qualitative post-test only
    5. Qualitative pre/post-test
    6. Cross-sectional survey
    7. Repeated cross-sectional surveys
    8. Mixed methods
    9. Program monitoring data
    10. Policy review
12. Details if ‘mixed-methods’ or if ‘policy review’ (i.e. framework/method utilized)
13. HIV-related outcomes (including whether measures were validated)
14. Summary of Intervention
15. Quantitative results
16. Qualitative results
17. Policy review results
18. UNAIDS human rights program categories
    1. HIV-related legal services
    2. Monitoring and reforming laws, policies and regulations
    3. Legal literacy
    4. Sensitization of lawmakers and law enforcement agents
    5. Training for health care providers on human rights and medical ethics related to HIV
    6. Multiple approaches
19. Multiple approaches (yes/no)
20. Number of approaches
21. Human Rights Based Approach (HRBA) principles
    1. Empowerment
    2. Non-discrimination
    3. Participation
    4. Accountability
    5. Linkages
    6. Multiple
22. Details if ‘multiple’ (e.g. list out all)
23. Attributes of the right to health
    1. Availability
    2. Accessibility
    3. Acceptability
    4. Quality
24. Multiple attributes of the right to health addressed (yes/no)
25. Number of attributes of the right to health addressed
26. Attention to human rights (implicit or explicit)
27. Notes
